# Supplementary material for: An observational, multicenter, registry-based cohort study of Turkish Neonatal Society in neonates with Hypoxic ischemic encephalopathy
Source: PLoS One. 2023 Dec 14;18(12):e0295759. doi: 10.1371/journal.pone.0295759 (PMC10721008; doi:10.1371/journal.pone.0295759)
Supplement: Attachment — Submitted filename: Response to Reviewers.docx [file pone.0295759.s002.docx]

Dear Editor and Reviewers,

We appreciate the time you and the reviewers spent to review our paper and contribute valuable feedback. Your valuable and insightful comments inspired potential improvements to this current version. The authors have carefully considered the comments and have done our best to respond to each one. We hope that the manuscript will be able to live up to your high expectations following focused edits. Any further beneficial criticism will always be welcomed by the authors.

We have listed the point-to-point responses below, and added the manuscript with tracking changes.

Sincerely,

Corresponding author

Emel Okulu

**Response to Reviewers**

**Reviewer #1:**

**Comment:** Please improve the quality of the figures. It's hard to read the figures.

**Response:** The figures were revised and added.

**Comment:** In these HIE instances, were there any sex differences?

**Response:** There were no sex differences according to HIE stages. This analysis is present in Table 1.

**Comment:** For more thorough information, also provide the sex details and their results should be supplied. Please discuss any outcome if available.

**Response:** This information is present in results section under the heading ‘Data of infants ≥36 weeks GA according to grading of HIE’. The word ‘gender’ was changed to ‘sex distrubition’.

**Comment:** The long-term neurodevelopmental effects of newborns, which may be crucial to discuss before drawing any conclusions about when TH should start, are important aspects. Please modify the conclusion and discuss this in details.

**Response:** Long-term follow-up is essential for this population. The conclusion revised as you recommended.

**Reviewer #2:** 
**Comment:** Minor corrections:- line 366: number missing - value: "mild HIE: ......median"; please added; - line 508: need to be rewritten; a new term for HI "trauma" is needed?
**Response:** The missing value added and ‘trauma’ changed as brain damage.

**Comment:** It is interesting that a postpartum event (probably in the first hours after birth), most likely defined as Apparent Life-Threatening Event, occurred in 10.3% of newborns treated with hypothermia. Do you have an explanation for this percentage of postpartum events underlying HIE?

**Response:** This percentage may be high to due to meconium aspiration syndrome which was found as the most common cause for postpartum asphyxia.

**Comment:** How can you explain findings in your study that only severe HIE is associated with
dysfunction of one organ system, when, we know that even mild and moderate hypoxia
is accompanied by impairment of organ systems; including the bone marrow.

**Response:** In our study, it was found that it was not only the infants with severe HIE who had impairment of organ systems. Almost all infants (90.7%) with severe HIE, 71.8% of moderate HIE and 34.6% of mild HIE had at least one problem. This information is present in both text and Table 2.

**Comment:** Before drawing conclusions about the differences between groups based on MRI and aEEG, would it make sense to analyse their neurodevelopmental outcomes at 24 months of age? Because HI is also a long-term process; when analysing the effectiveness of hypothermia, we do not take into account the tertiary phase of HE brain damage, which continues for a long time after the acute event. Do you have any data about neurodevelopmental outcome of children treated with TH in newborn period?

**Response:** Thank you for your comment, you are absolutely right. Unfortunately, we do not have the data about neurodevelopmental outcome of these children now, but we are planning another study to collect this data soon.

**Comment:** The same applies to the interpretation of TH in late preterm infants, please.

**Response:** We are planning another study to collect this data soon

**Comment:** Perhaps, a higher predictive value when comparing groups would be achieved if the brain magnetic resonance imaging will be assessed based on MRI scoring systems for HIE brain injury designed by Weeke and co-authors. I will suggest to use Weekes MRI scoring system as a validated and standardized tool for defining HI brain injury and as a marker for the analysis of two groups in your study.

**Response:** We thank for your recommendation. It has been shown that Weeke scoring system is a high importance to assess the impact of perinatal asphyxia on MR imaging.

It would be good to use this scoring system. We reviewed our data in this respect, but we could not adjust our data on the extension of injury used for scoring. Therefore, we regret that we could not complete this suggestion.

**Comment:** Your study highlights that the overall incidence of severe HIE has decreased; which is consistent with the results of other studies. Given the fact that your study includes only inborn patients, would the results be the same if you also included outborn neonates?

**Response:** The registry data contained information on the proportion of inborn and outborn infants at each participating center. Certain outborn infants were transferred to a participating center for one of the following three reasons: i. No TH device available, ii.The TH device is in use by another patient, iii. NICU capacity is full. It may be challenging and inaccurate to calculate the incidence by simply asking the number of deliveries from the sending hospital, so the only the incidence of HIE was calculated according to inborn HIE infants.

**Comment:** With the introduction of the national registry of newborns with HIE, did you also notice a difference in the treatment of such newborns before and after the introduction of the registry, such as achieving the same cooling temperature between different centres; unified neuromonitoring, unified imaging diagnostics and unified follow up?

**Response:** Obviously we don’t know that. Nonetheless, the centers are maintaining more comprehensive records of these patients for this registry and beyond.
